# Mitofusin 2 is required for preventing deoxynivalenol-induced porcine intestinal epithelial cell damage

**DOI:** 10.1186/s40104-025-01306-6

**Published:** 2025-12-23

**Authors:** Kan Xiao, Minfang Zhang, Qingqing Lv, Feifei Huang, Qilong Xu, Junjie Guo, Jiangchao Zhao, Huiling Zhu, Shaokui Chen, Yulan Liu

**Affiliations:** 1 Hubei Key Laboratory of Animal Nutrition and Feed Science, Wuhan Polytechnic University, Wuhan, People’s Republic of China; 2 College of Animal Science, South China Agricultural University, Guangzhou, People’s Republic of China

**Keywords:** Deoxynivalenol, Intestinal injury, Mfn2, Mitochondrial homeostasis, Piglets

## Abstract

**Backgrounds:**

Deoxynivalenol (DON) is an abundant environmental pollutant in feed, posing serious health hazards to animals. However, whether DON triggers an imbalance in mitochondrial fission/fusion and the underlying mechanisms involved remain poorly understood. Our aim was to clarify whether mitochondrial fission or fusion proteins participated in DON-caused intestinal damage in pigs.

**Methods:**

Firstly, two groups of weaning pigs were fed a basal diet, or basal diet supplemented with 4 mg DON/kg for 3 weeks. Additionally, another two groups of weaning pigs were given an oral gavage with 2 mg/kg body weight DON or an equivalent amount of normal saline. In addition, the involvement of mitochondrial fission or fusion proteins in DON-induced intestinal damage was further verified in intestinal porcine epithelial cell line (IPEC-1) by overexpressed plasmids of dynamin related protein 1 (Drp1) and mitofusin 2 (Mfn2) which were determined by animal studies. Finally, a mitochondrial fusion promotor M1 was used in IPEC-1 cells to explore the role of Mfn2 in DON-induced intestinal damage.

**Results:**

Dietary DON caused jejunal damage and inflammation, reduced intestinal Drp1, mitofusin 1 (Mfn1) and Mfn2, and induced cell apoptosis. DON gavage also impaired jejunal structure and led to decreased Drp1 and Mfn2, and increased cell apoptosis. Moreover, DON challenge also resulted in cell damage and mitochondrial dysfunction, accompanied by abnormal protein expression of mitochondrial fission/fusion proteins and increased cell apoptosis in IPEC-1 cells. Subsequently, Mfn2, but not Drp1 overexpression plasmid restored mitochondrial fission/fusion protein expression, suppressed cell apoptosis, mitigated cell damage and mitochondrial dysfunction in IPEC-1 cells after DON challenge. Finally, M1 alleviated DON-induced reduction of Mfn2 protein and cell apoptosis, rescued mitochondrial dysfunction, barrier function impairment and cell damage.

**Conclusions:**

Overall, our study demonstrates that DON exposure triggers Mfn2 protein dysregulation, which in turn mediates DON-induced intestinal epithelial damage in piglets.

**Supplementary Information:**

The online version contains supplementary material available at 10.1186/s40104-025-01306-6.

## Introduction

Deoxynivalenol (DON) is one of the most prevalent mycotoxins, which primarily generated by *Fusarium graminearum* and *Fusarium culmorum* [1]. The extensive contamination and high toxicity of DON present considerable food safety risks for livestock [2]. As the first defense against external contaminants, the gut is directly exposed to high DON concentrations following continuous ingestion [3]. DON-contaminated feed usually induces diverse adverse effects in farm animals, ranging from feed refusal, vomiting, and diarrhea to potentially lethal outcomes [4]. While different animal species exhibit varying degrees of DON sensitivity, swine are particularly susceptible to its toxic effects [5]. In piglets, repeated DON exposure has been linked to intestinal damage, immune suppression, growth retardation, and reproductive dysfunction [6, 7]. Although the general mechanisms underlying DON-induced intestinal toxicity are well-documented, the relationship between DON and mitochondrial fission and fusion in porcine intestinal models remain insufficiently characterized.

Mitochondrial fission and fusion are critical for sustaining mitochondrial structure and function when cells suffer from adverse external stresses [8]. Mitochondrial fusion helps to attenuate stress by integrating damaged mitochondria contents as a complementation, which is executed by mitofusin 1 (Mfn1), Mfn2 or optic atrophy protein 1 (Opa1) [9, 10]. Conversely, mitochondrial fission is essential not only for mitochondrial proliferation but also for quality control-eliminating damaged mitochondria and initiating apoptosis under severe stress. This process is regulated by dynamin-related protein 1 (Drp1) and fission protein 1 (Fis1) [11, 12]. Mitochondrial fusion/fission is usually in a state of dynamic equilibrium under physiological conditions and their disturbances are usually implicated in several diseases such as heart and brain damage [13–16]. Emerging evidence highlights the critical role of mitochondrial dynamics in intestinal homeostasis and inflammatory bowel disease (IBD). A recent study revealed that impaired mitochondrial fusion in intestinal epithelial cells of IBD patients contributed to chronic intestinal inflammation, as demonstrated in murine models [17]. Additionally, another investigation identified that excessive mitochondrial fission, mediated by Drp1 and Fis1, disrupts mucosal repair by impairing butyrate metabolism in colonic epithelial cells from ulcerative colitis patients and in dextran sulfate sodium-induced colitis of mice [18]. However, it remains unknown whether abnormal mitochondrial fission/fusion is involved in intestinal injury after DON exposure in human or livestock.

Therefore, the aim of this research was to investigate whether mitochondrial fission/fusion proteins were involved in DON-caused intestinal damage. Weaning pigs, which are particularly susceptible to DON [19], were chosen as the experimental model to investigate whether DON-induced intestinal injury is associated with dysregulation of mitochondrial fission/fusion proteins. In pig production, DON is predominantly ingested by animals through repeated exposure via contaminated feed. In the laboratory, we established a stable model of acute intestinal injury in piglets by administering large doses of DON via oral gavage. Our results may provide new perspectives for preventing or treating DON exposure-induced intestinal disorders.

## Materials and methods

### Animal, diet and experimental design

The Animal Care and Use Committee of Wuhan Polytechnic University approved these animal trials and the ethics approval codes were as follows: EM20221216006 and EM20230708003. Weaned piglets (Duroc × Large White × Landrace; barrows; initial body weight (BW) of 7.05 ± 0.71 kg; 28 days of age) were purchased from Aodeng Agriculture and Animal Husbandry Technology Co., Ltd. (Hubei, China) and housed in 1.80 m × 1.10 m pens and maintained in an environmentally controlled physiology room with free access to feed and water. During the experimental period, pigs were fed a diet formulated in accordance with nutrient requirements of swine (NRC 2012) [20].

In Exp. 1, 12 weaned pigs were randomly allocated to two groups including control group and dietary DON group (repeated DON exposure). There were 6 piglets per group. Pigs in DON group fed a basal diet supplemented with 4 mg/kg DON which was cultivated from *Fusarium graminae* W3008 based on Wang et al. [21]. The dosage of DON in diet was chosen based on previous studies [21, 22]. The DON concentrations in basal diet and DON-contaminated diet were 0.3 mg/kg and 4 mg/kg, respectively. The experiment lasted for 21 d. At the end of this experiment, blood sample were collected and then all piglets (56 days of age) were anesthetized by intramuscular injection with Zoletil^®^ 50 (10 mg/kg BW) to euthanasia for jejunal samples.

In Exp. 2, another 12 weaned pigs were randomly assigned to two groups containing control group and DON gavage group (acute DON exposure). The piglets in DON gavage group were given an oral gavage of 2 mg/kg BW DON (Pribolab, Qingdao, China) after fasted 12 h. The gavage dosage of DON and the time point were selected based on our preliminary study. Pigs in control group received an equivalent amount of normal saline. After 6 h post gavage, all pigs were sacrificed for sample collection in the same method as Exp. 1.

In Exp.3, IPEC-1 cells were maintained in DMEM-F12 medium as previously described [23]. The cells were exposed to different concentrations of DON (Sigma-Aldrich, MO, USA; 0, 0.2, 0.5, 1, and 2 μg/mL) for 48 h. Following treatment, both cells and culture supernatants were harvested for further analysis.

To investigate the role of mitochondrial dynamics, IPEC-1 cells were transfected with either Drp1 (NCBI RefSeq: XM_021092060.1) or Mfn2 (NCBI RefSeq: XM_021095349.1) overexpression (OE) plasmids (constructed by Wuhan Miaoling Biology). Transfected cells were then treated with or without 0.5 μg/mL DON for 48 h, after which cells and supernatants were collected. This DON dosage was selected based on previous results. Additionally, to assess the effects of mitochondrial fusion, IPEC-1 cells were pretreated with the mitochondrial fusion promoter M1 (10 nmol/L) or vehicle control before exposure to 0.5 μg/mL DON. Cells and supernatants were subsequently harvested for analysis.

### Intestinal morphology

After fixation, jejunal segments were prepared in accordance with traditional paraffin-embedding techniques. Jejunal tissues were cut into a 5-μm thickness slice and dyed with hematoxylin and eosin (HE) reagents. The villus height and crypt depth were visualized by a microscope (Olympus CX31, Olympus Corporation, Tokyo, Japan). The villus height:crypt depth ratio was calculated according to villus height and crypt depth. Ten well-oriented and intact villi were selected for measurement for every slice.

### Inflammatory indexes

The concentrations of TNF-α (CSE0005-096, 4 A Biotech, Beijing, China) and IL-6 (CSE0006-096, 4 A Biotech) in serum were quantified using commercial ELISA kits according to the manufacturer’s protocols. Briefly, after preparation of working solutions, standards and test samples were loaded into the microplate wells, followed by incubation with biotinylated detection antibody at room temperature. Subsequently, enzyme conjugate working solution was added and incubated, followed by chromogenic substrate reaction for 20 min. The reaction was terminated by stop solution, and the optical density at 450 nm (OD_450_) was immediately measured using a microplate reader (FLx800, Bio-Tek Instruments Inc., Winooski, VT, USA). Cytokine concentrations were calculated based on standard curves generated for each assay.

Neutrophil infiltration density was histologically quantified from HE-stained sections under a light microscope (Olympus CX31, Olympus Corporation, Tokyo, Japan) at 400 × magnification.

### Cell viability

Cell viability was assessed using Cell Counting Kit-8 assays (C0038, Beyotime Biotechnology, Shanghai, China). After DON treatment for 48 h, CCK-8 reagent was added to each well and incubated for 1 h. Absorbance was measured at 450 nm using a microplate reader (BioTek Instruments, Winooski, VT, USA).

### Lactate dehydrogenase (LDH) activity

LDH activity in cell supernatant was measured using the corresponding LDH kits (A020-1, Nanjing Jiancheng, Jiangsu, China) according to the manufacturer’s instructions. After DON treatment for 48 h, absorbance was quantified at 490 nm using a microplate reader (BioTek Instruments, Winooski, VT, USA).

### Barrier function

IPEC-1 cells grew on collagen-coated permeable polycarbonate filters (Corning, NY, USA) with a density of 1.5 × 10^5^ cells/well. Transepithelial electrical resistance (TEER) was monitored using a Millicell-Electrical Resistance System 2 (Millipore, USA) and determined every 24 h according to our previous experiment [21]. Following 10 days of cell culture, varying concentrations of DON ( 0, 0.2, 0.5, 1, and 2 μg/mL) were administered to the culture chambers for 48 h. To assess monolayer permeability, fluorescein isothiocyanate-labeled 4 kDa dextran (FD4; Sigma-Aldrich, MO, USA) was added to the apical chamber, and its transport to the basolateral chamber was quantified using our established protocol [21].

### Mitochondrial membrane potential (MMP)

MMP was assessed using JC-1 fluorescent probe (40705ES03, Shanghai Yeasen Biotechnology Co., Ltd., Shanghai, China). Following the manufacturer’s protocol, IPEC-1 cells were incubated with JC-1 working solution. Fluorescence intensities (red: 590 nm, green: 530 nm) were quantified using a microplate reader (BioTek Instruments Inc., USA). MMP was expressed as the ratio of red to green fluorescence intensity, reflecting mitochondrial depolarization.

### Real-time PCR

Total RNA was extracted from IPEC-1 cells by TRIzol reagent (TaKaRa Biotechnology (Dalian) Co., Ltd., Dalian, China) based on the procedure of manufacturer’s recommendations. After removing genomic DNA, cDNA was synthesized using a reverse transcription kit. Real-time PCR was performed using a SYBR Premix Ex Taq™ kit (TaKaRa) on a 7500 Real-Time PCR System (Applied Biosystems). Primer sequences are provided in Table S1. Relative gene expression was calculated by the 2^−ΔΔCt^ method based on Livak and Schmittgen [24]. Expression of target genes was normalized to *GAPDH*.

### Western blot analysis

Total protein was extracted from jejunal tissues or IPEC-1 cells using lysis buffer. Protein concentrations were quantified using a BCA assay kit (Thermo Fisher Scientific, USA). Equal amounts of protein were separated by SDS-PAGE and subsequently transferred onto PVDF membranes. After blocking with 5% non-fat milk, the membranes were incubated overnight with the following primary antibodies: rabbit anti-Drp1 (1:1,000, #Ab154879, Abcam), rabbit anti-Fis1 (1:1,000, #10956-1-AP, Proteintech), rabbit anti-Opa1 (1:1,000, #NB110-55290ss, Novus), rabbit anti-Mfn1 (1:1,000, #PA5-44826, Invitrogen), mouse anti-Mfn2 (1:1,000, #ab56889, Abcam), and mouse anti-β-actin (1:2,000, #4970s, CST). The next day, membranes were incubated with horseradish peroxidase-conjugated secondary antibodies (1:5,000) for 1 h at room temperature. Protein bands were visualized using an enhanced chemiluminescence kit (Amersham Biosciences, UK) and quantified using Quantity One^®^ software (Bio-Rad Laboratories, USA). The expression levels of target proteins were normalized to β-actin and presented as relative band intensities.

### Statistical analysis

For the animal trials, data were analyzed by independent-sample *t*-tests using SAS (Cary, NC, USA). In the in vitro trial, data from different DON doses were analyzed by one-way ANOVA. Data from Drp1 (Mfn2) OE and M1 were analyzed by two-way ANOVA for a 2 × 2 factorial design. When significant interactions occurred, multiple comparison tests were performed using Duncan's multiple comparisons. All data are expressed as mean ± standard error. *P* ≤ 0.05 was considered statistically significant, and 0.05 < *P* ≤ 0.1 were considered a trend. All data were plotted using GraphPad Prism Version 8 software (Graphpad software, LLC, San Diego, USA).

## Results

### Effects of DON on intestinal structure in piglets

**Fig. 1:**
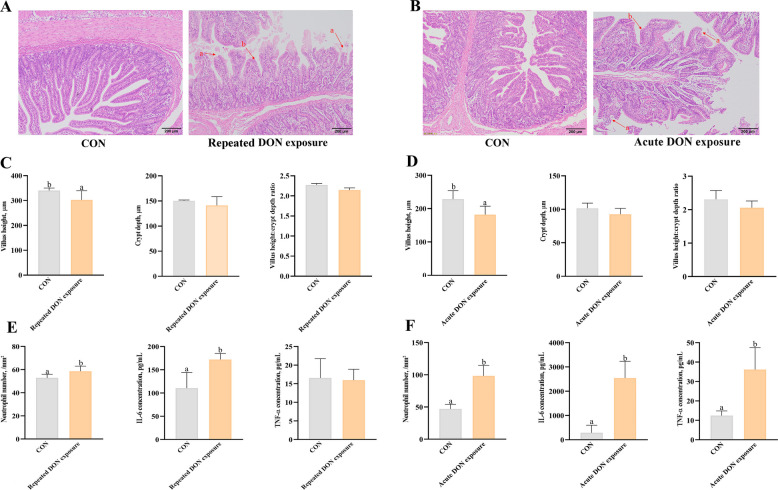
Effects of DON on intestinal structure in piglets. **A** Representative photomicrographs of villus (HE-stained) in jejunum from control piglets fed basal diet and piglets fed 4 mg/kg DON-contaminated diet. Arrows indicate lifting of epithelium at the tip of the villus, and villus atrophy. The magnification was 40 ×. **B** Representative photomicrographs of villus (HE-stained) in jejunum from control piglets, and piglets orally gavaged with 2 mg/kg DON. **C** Villus height, crypt depth and ratio of villus height to crypt depth of jejunum in response to dietary DON. **D** Villus height, crypt depth and ratio of villus height to crypt depth of jejunum in response to 2 mg/kg DON gavage. **E** The neutrophil density and serum inflammatory cytokines in response to dietary DON. **F** The neutrophil density and serum inflammatory cytokines in response to 2 mg/kg DON gavage. Values are means ± SEM, *n* = 6. ^a,b^Means without a common letter differ (*P* < 0.05). DON, deoxynivalenol; TEM, transmission electron microscope

The intestinal tract is directly exposed to high concentrations of DON, making it a primary target for toxin-induced damage. We first measured the jejunal structure. As depicted in Fig. 1A, dietary DON (repeated DON exposure) caused intestinal morphologic changes such as epithelium lifting at the tip of the villus, and villus atrophy. Dietary DON reduced villus height and villus height: crypt depth ratio (*P* < 0.05) in jejunum (Fig. 1C). Further analysis showed that dietary DON increased the neutrophil infiltration density in jejunum and increased IL-6 content in serum of pigs (Fig. 1E).

Additionally, acute DON oral gavage (acute DON exposure) resulted in abnormal jejunal structure as exhibited by epithelium lifting at the tip of the villus, villus atrophy and hemorrhage in lamina propria (Fig. 1B). DON gavage lowered villus height (*P* < 0.05) in jejunum of piglets (Fig. 1D). DON gavage also significantly elevated neutrophil infiltration in jejunal tissues (*P* < 0.05) and upregulated serum concentrations of pro-inflammatory cytokines IL-6 and TNF-α (*P* < 0.05) in piglets (Fig. 1F).

### Effects of DON on mitochondrial fission/fusion protein expression and cell apoptosis in jejunum of piglets

**Fig. 2:**
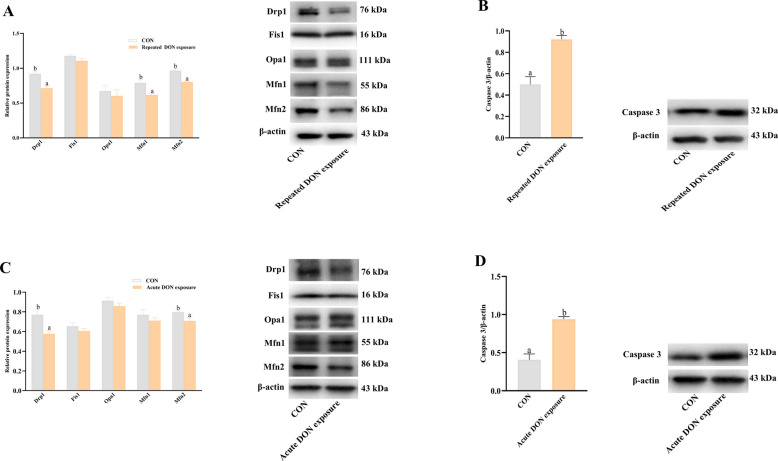
Effects of DON on intestinal mitochondrial fission/fusion dynamic and cell apoptosis in jejunum of piglets. **A** Mitochondrial fission/fusion protein expression and representative bands in jejunum from control piglets fed basal diet and piglets fed 4 mg/kg DON-contaminated diet. **B** Caspase 3 protein expression and representative band intensity quantification in jejunum from control piglets fed basal diet and piglets fed 4 mg/kg DON-contaminated diet. **C** Mitochondrial fission/fusion proteins and representative band intensity quantification in jejunum from control piglets orally gavaged with saline and piglets orally gavaged with 2 mg/kg DON. **D** Caspase 3 protein expression and representative band intensity quantification in jejunum from control piglets orally gavaged with saline and piglets orally gavaged with 2 mg/kg DON. Values are means ± SEM, *n* = 6. ^a,b^Means without a common letter differ (*P* < 0.05). Drp1, dynamin related protein 1; DON, deoxynivalenol; Fis1, fission protein 1; Mfn2, mitofusin 2; Opa1, optic atrophy protein 1

To investigate the effect of DON on intestinal mitochondrial fission/fusion dynamics, we measured protein expression of mitochondrial fission/fusion proteins in jejunum of piglets. As shown in Fig. 2, dietary DON significantly decreased protein abundance of Drp1, Mfn1, and Mfn2 (*P* < 0.05) in jejunum (Fig. 2A). Consistent with repeated dietary exposure to DON, DON gavage also downregulated protein expression of Drp1 and Mfn2 (*P* < 0.05) in jejunum of piglets (Fig. 2C).

Mitochondrial fission/fusion imbalance is closely associated with cell apoptosis. We next explored if DON exposure could lead to intestinal cell apoptosis in piglets. As depicted in Fig. 2B, dietary DON increased jejunal protein expression of caspase-3 (*P* < 0.05), a marker protein of cell apoptosis. Similarly, DON gavage also upregulated protein abundance of caspase-3 (*P* < 0.05) (Fig. 2D).

### Effects of DON on cell injury, mitochondrial fission/fusion imbalance and cell apoptosis in IPEC-1 cells

**Fig. 3:**
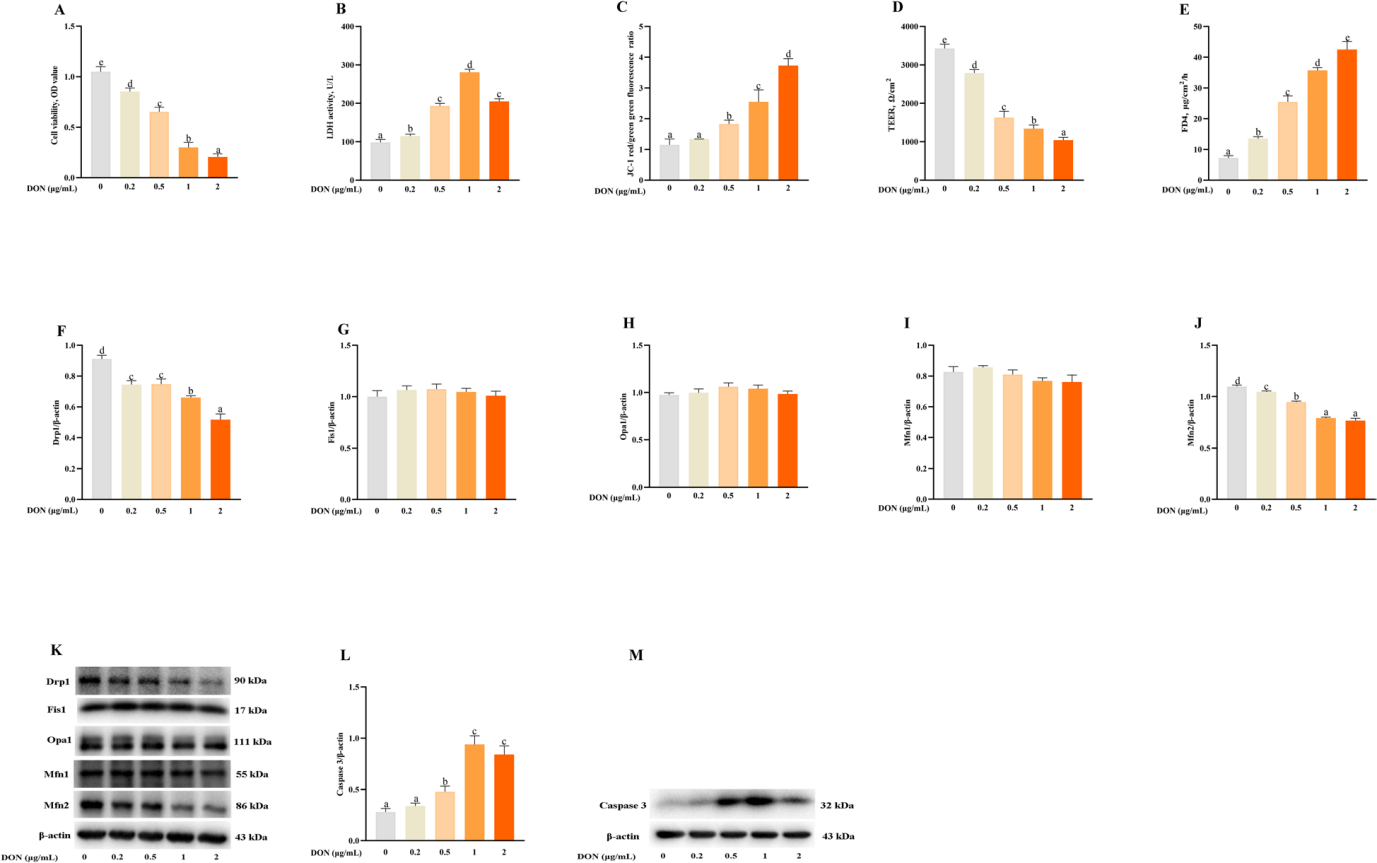
Effects of DON on cell injury, abnormal mitochondrial fission/fusion dynamic and cell apoptosis in IPEC-1 cells. Cells were treated with different concentrations (0, 0.2, 0.5, 1 and 2 μg/mL) of DON for 48 h. **A** Cell viability. **B** LDH. **C** MMP. **D**–**J** Mitochondrial fission/fusion proteins expression. **K** Representative bands of mitochondrial fission/fusion proteins. **L** Caspase 3 protein expression. **M** Representative band of caspase 3 protein expression. Values are means ± SEM, *n* = 8. ^a−e^Means without a common letter differ (*P* < 0.05). Drp1, dynamin related protein 1; DON, deoxynivalenol; Fis1, fission protein 1; IPEC, intestinal porcine epithelial cells; LDH, lactate dehydrogenase; MMP, mitochondrial membrane potential, Mfn2, mitofusin 2; Opa1, optic atrophy protein 1

To further verify the role of DON on intestinal cell damage, mitochondrial fission/fusion imbalance and cell apoptosis, we challenged IPEC-1 cells with DON. As shown in Fig. 3A–C, DON treatment reduced cell viability, raised LDH activity and depressed mitochondrial membrane potential (MMP) (*P* < 0.05) of IPEC-1 cells. Moreover, DON reduced TEER value and increased FD4 permeability (*P* < 0.05) in IPEC-1 cells (Fig. 3D and E). Furthermore, consistent with the results of piglets, DON challenge decreased Drp1 and Mfn2 protein expression (*P* < 0.05) in IPEC-1 cells (Fig. 3F–K). DON challenge also improved caspase-3 protein abundance (*P* < 0.05) in IPEC-1 cells (Fig. 3L–M).

### Effects of Drp1 and Mfn2 overexpression on mitochondrial dysfunction and cell injury after DON challenge in IPEC-1 cells

**Fig. 4:**
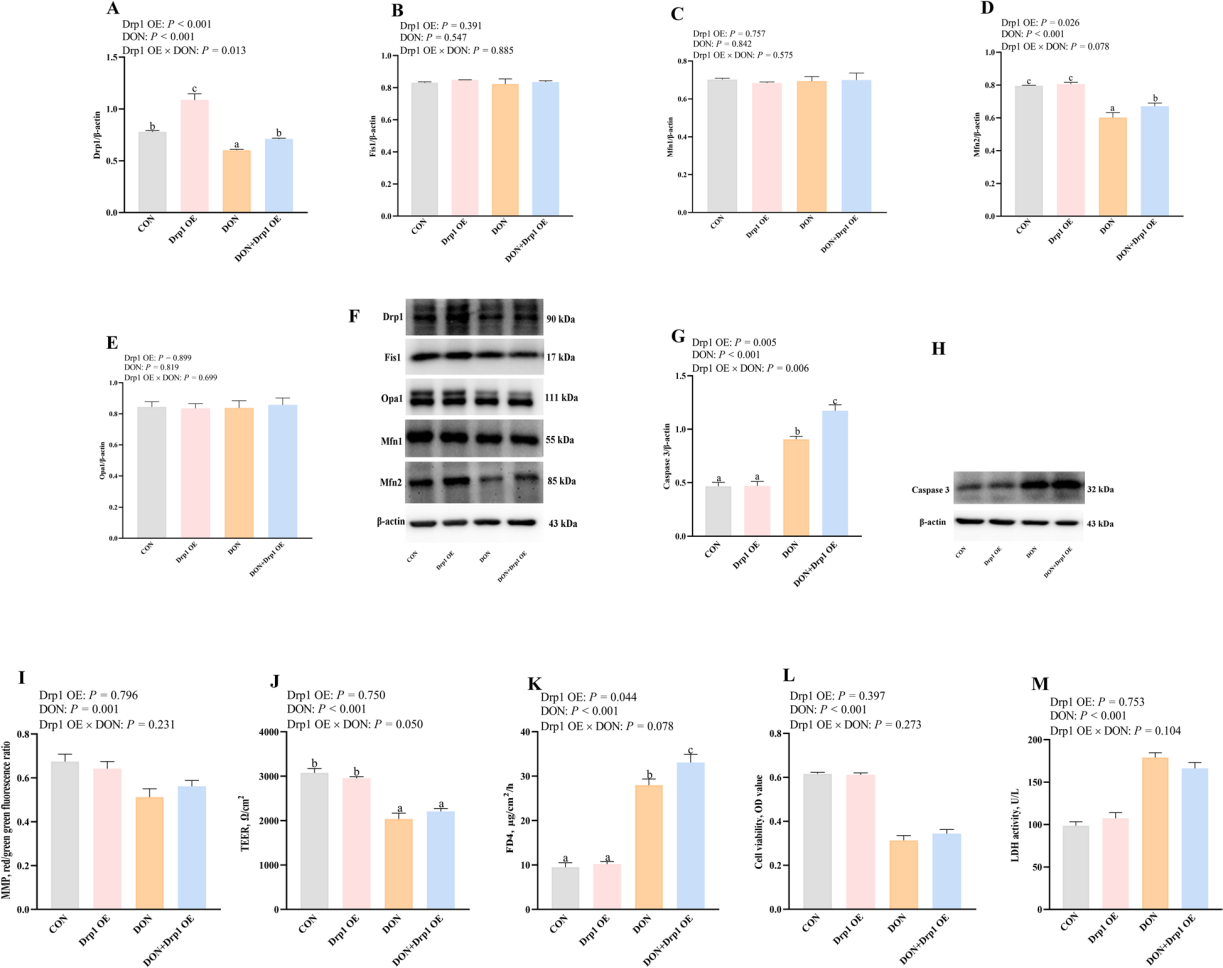
Effects of Drp1 overexpression on mitochondrial dysfunction and cell injury after DON challenge in IPEC-1 cells. Cells were first pretreated with overexpression plasmid of Drp1 or Mfn2 for 24 h, and then challenged with DON for 48 h. **A**–**E** Mitochondrial fission/fusion protein expression after Drp1 overexpression plasmids treatment. **F** Representative bands of mitochondrial fission/fusion proteins after Drp1 overexpression plasmids treatment. **G** Caspase 3 protein expression after Drp1 overexpression plasmid treatment. **H** Representative band of caspase 3 protein expression after Drp1 overexpression plasmid treatment. **I** MMP after Drp1 overexpression plasmid treatment. **J** TEER after Drp1 overexpression plasmid treatment. **K** FD4 after Drp1 overexpression plasmid treatment. **L** Cell viability after Drp1 overexpression plasmid treatment. **M** LDH after Drp1 overexpression plasmid treatment. Values are means ± SEM, *n* = 8. ^a−d^Means without a common letter differ (*P* < 0.05). Drp1, dynamin related protein 1; DON, deoxynivalenol; Fis1, fission protein 1; IPEC, intestinal porcine epithelial cells; Mfn2, mitofusin 2; OE, overexpression; Opa1, optic atrophy protein 1; LDH, lactate dehydrogenase; MMP, mitochondrial membrane potential; FD4, fluorescein isothiocyanate-labeled dextran 4 kDa; TEER, transepithelial electrical resistance

**Fig. 5:**
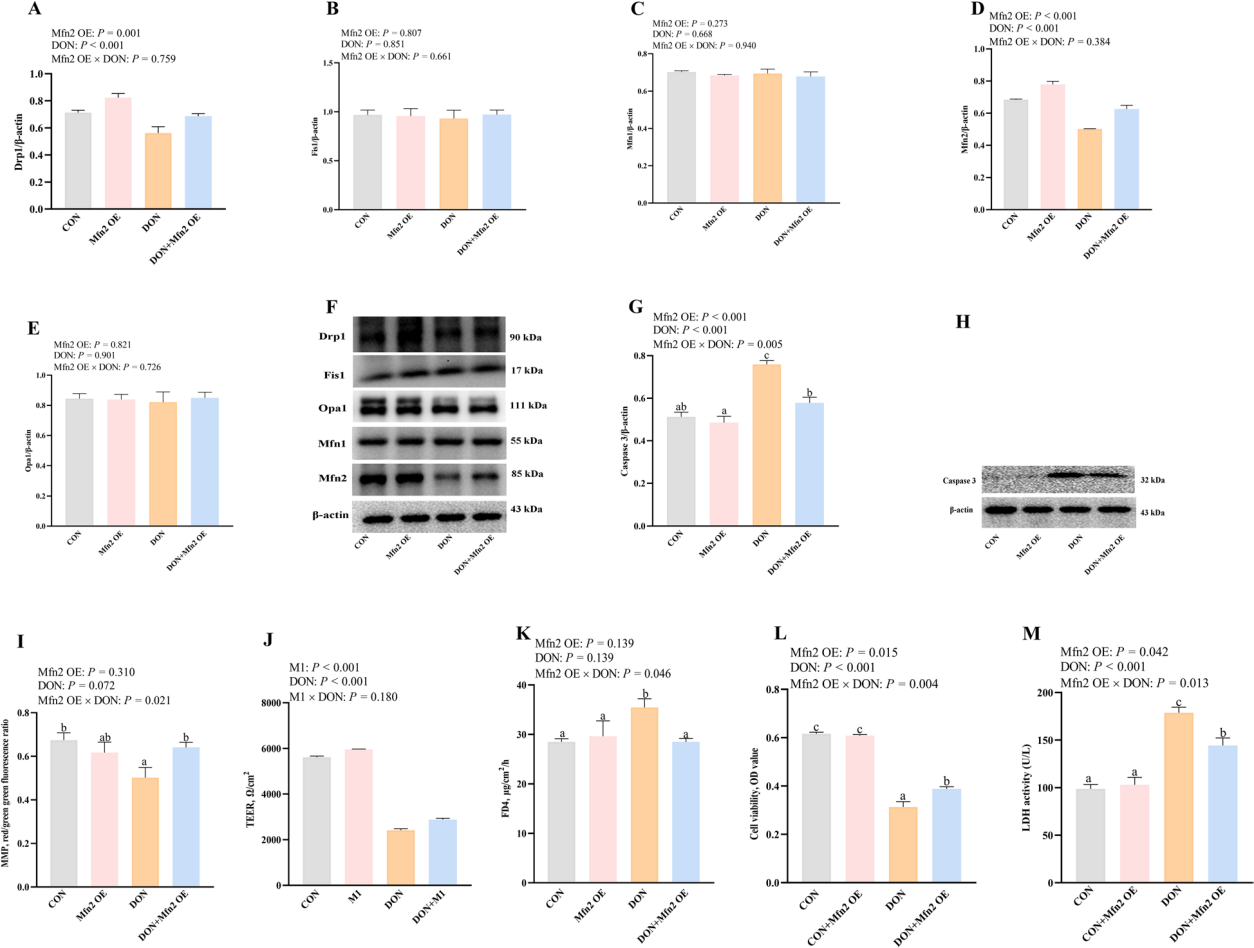
Effects of Mfn2 overexpression on mitochondrial dysfunction and cell injury after DON challenge in IPEC-1 cells. Cells were firstly pretreated with overexpression plasmid of Drp1 or Mfn2 for 24 h, and then challenged with DON for 48 h. **A**–**E** Mitochondrial fission/fusion protein expression after Drp1 overexpression plasmids treatment. **F** Representative bands of mitochondrial fission/fusion proteins after Drp1 overexpression plasmids treatment. **G** Caspase 3 protein expression after Drp1 overexpression plasmid treatment. **H** Representative band of caspase 3 protein expression after Drp1 overexpression plasmid treatment. **I** MMP after Drp1 overexpression plasmid treatment. **J** TEER after Drp1 overexpression plasmid treatment. **K** FD4 after Drp1 overexpression plasmid treatment. **L** Cell viability after Drp1 overexpression plasmid treatment. **M** LDH after Drp1 overexpression plasmid treatment. Values are means ± SEM, *n* = 8. ^a−d^Means without a common letter differ (*P* < 0.05). Drp1, dynamin related protein 1; DON, deoxynivalenol; Fis1, fission protein 1; IPEC, intestinal porcine epithelial cells; Mfn2, mitofusin 2; OE, overexpression; Opa1, optic atrophy protein 1; LDH, lactate dehydrogenase; MMP, mitochondrial membrane potential; FD4, fluorescein isothiocyanate-labeled dextran 4 kDa; TEER, transepithelial electrical resistance

To further explore whether abnormal mitochondrial fission or fusion proteins contributed to DON-caused intestinal damage and mitochondrial dysfunction, Drp1 or Mfn2 overexpression plasmids were used. The overexpression efficiency was shown in Fig. S1. Drp1 or Mfn2 overexpression upregulated protein expression of Drp1 and Mfn2 (*P* < 0.05) after DON challenge, respectively (Figs. 4A–F and 5A–F). There was a Drp1 OE × DON interaction observed (*P* < 0.05) for Drp1 protein expression in which Drp1 OE increased the protein expression of Drp1 in DON-challenged cells (Fig. 4A). There was a trend for Drp1 OE × DON interaction observed (*P* = 0.078) for Mfn2 protein expression in which Drp1 OE increased Mfn2 protein expression in DON-challenged cells, whereas the protein expression of Mfn2 did not differ in non-DON challenged cells (Fig. 4D).

Furthermore, we verified if Drp1 or Mfn2 overexpression has positive effects on DON-induced cell apoptosis. DON exposure increased the caspase 3 protein expression in IPEC-1 cells (Figs. 4G, H and 5G, H). There was a Drp1 OE × DON interaction observed (*P* < 0.05) for caspase 3 in which Drp1 OE increased the protein expression of caspase 3 in DON-challenged cells, whereas the protein expression of caspase 3 did not differ in non-DON challenged cells (Fig. 4G and H). There was a Mfn2 OE × DON interaction observed (*P* < 0.05) for caspase 3 protein expression in which Mfn2 OE decreased caspase 3 protein expression in DON-challenged cells, whereas the protein expression of caspase 3 did not differ in non-DON challenged cells (Fig. 5G and H). These data showed that only Mfn2, but not Drp1 overexpression prevented the increase of caspase-3 protein expression after DON exposure, which suggested that promoting mitochondrial fusion protein Mfn2 inhibited DON-induced cell apoptosis in IPEC-1 cells.

We next investigated the role of Drp1 or Mfn2 in DON-caused mitochondrial dysfunction and cell injury. As depicted in Figs. 4I–M and 5I–M, Mfn2 overexpression, but not Drp1 overexpression, suppressed the decrease of MMP and improved cell barrier integrity (*P* < 0.05) after DON exposure. Mfn2 overexpression also increased cell viability and reduced LDH activity (*P* < 0.05) in IPEC-1 cells after DON challenge. There were Mfn2 OE × DON interactions (*P* < 0.05) observed for MMP, FD4, cell viability and LDH activity in which Mfn2 OE increased MMP and cell viability, and decreased FD4 flux and LDH activity in DON-challenged cells, whereas these indexes did not differ in non-DON challenged cells (Fig. 5I–M).

### Effects of M1 on mitochondrial dysfunction and cell injury after DON challenge in IPEC-1 cells

**Fig. 6:**
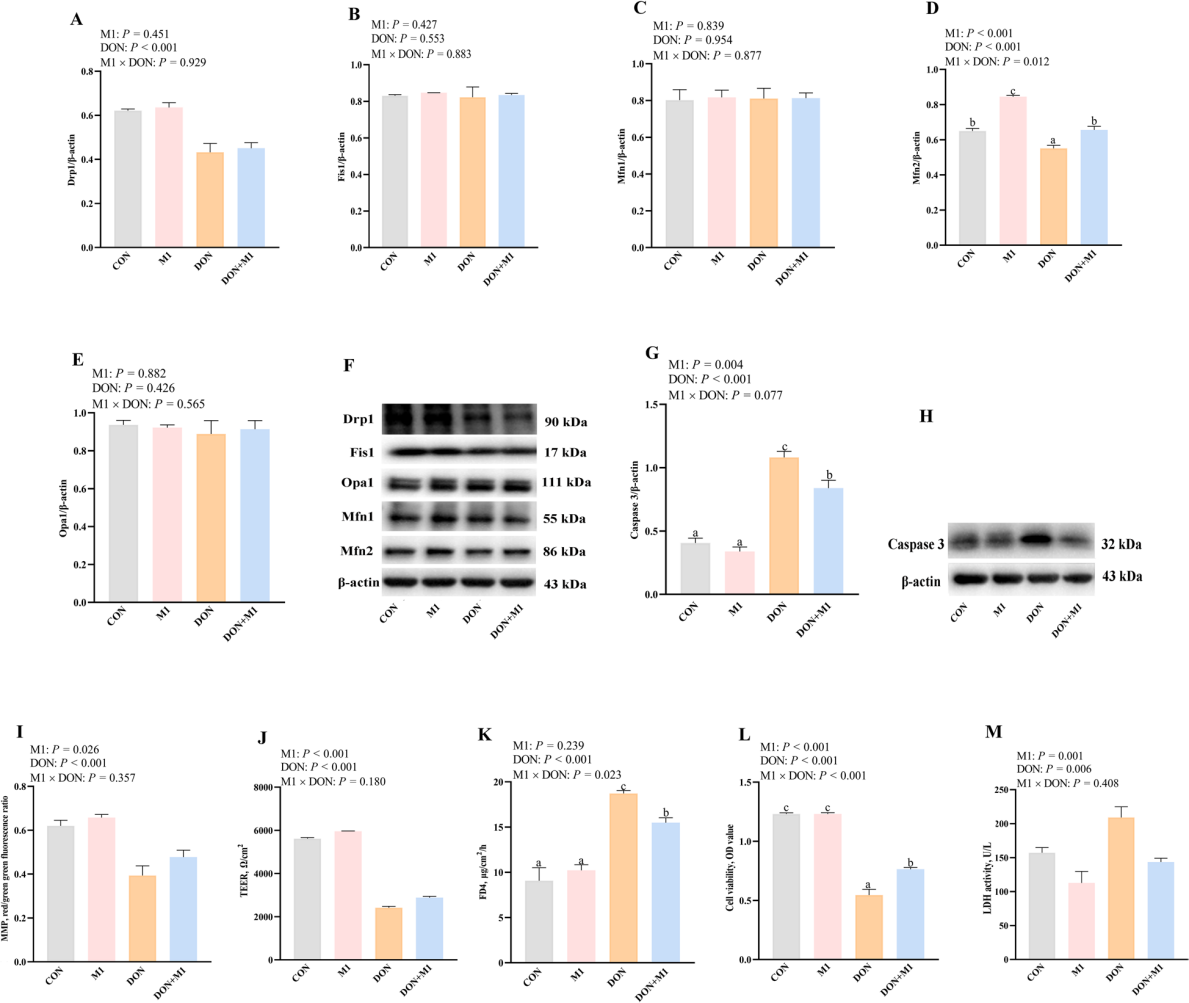
Effects of M1 on mitochondrial dysfunction and cell injury after DON challenge in IPEC-1 cells. **A**–**E** Representative band intensity quantification of mitochondrial fission/fusion proteins after M1 treatment. **F** Caspase 3 protein expression after M1 treatment. **G** Representative band intensity quantification of caspase 3 protein expression after M1 treatment. **H** Caspase 3 protein expression after M1 treatment. **I** MMP of IPEC-1 cells after M1 treatment. **J** TEER of IPEC-1 cells after M1 treatment. **K** FD4 of IPEC-1 cells after M1 treatment. **L** Cell viability of IPEC-1 cells after M1 treatment. **M** LDH of IPEC-1 cells after M1 treatment. Values are means ± SEM, *n* = 8. ^a−d^Means without a common letter differ (*P* < 0.05). Drp1, dynamin related protein 1; DON, deoxynivalenol; Fis1, fission protein 1; IPEC, intestinal porcine epithelial cells; Mfn2, mitofusin 2; Opa1, optic atrophy protein 1; LDH, lactate dehydrogenase; MMP, mitochondrial membrane potential; FD4, fluorescein isothiocyanate-labeled dextran 4 kDa; TEER, transepithelial electrical resistance

To further explore the role of Mfn2 in DON-induced intestinal damage and mitochondrial disorders, we used mitochondrial fusion promoter M1 to pretreat IPEC-1 cells before DON challenge. M1 improved protein abundance of Mfn2 after DON exposure. There was a M1 × DON interaction (*P* < 0.05) observed for Mfn2 protein expression in which M1 increased Mfn2 protein expression in DON-challenged cells and non-DON-challenged cells (Fig. 6A–F).

We further investigated whether reduced Mfn2 was involved in DON-induced intestinal apoptosis. M1 decreased protein abundance of caspase-3 (*P* < 0.05) after DON exposure in IPEC-1 cells (Fig. 6G and H). There was a trend for M1 × DON interaction (*P* = 0.077) observed for caspase 3 protein expression in which M1 decreased caspase 3 in DON-challenged cells, however, caspase 3 protein expression did not differ in non-DON challenged cells (Fig. 6G and H).

Moreover, we explored the role of Mfn2 in preventing mitochondrial dysfunction and cell damage. As depicted in Fig. 6I–M, M1 increased MMP, TEER and cell viability (*P* < 0.05), and decreased LDH activity (*P* < 0.05) in IPEC-1 cells after DON exposure. There were M1 × DON interactions (*P* < 0.05) observed for FD4 and cell viability in which M1 increased cell viability and decreased FD4 flux in DON-challenged cells, whereas FD4 and cell viability did not differ in non-DON challenged cells (Fig. 6I–M).

## Discussion

In this study, we provide in vivo and in vitro evidence that decreased mitochondrial fission/fusion protein expression is accompanied by DON-induced intestinal injury in piglets. Rescuing mitochondrial fusion protein Mfn2 by overexpression or promotor, but not mitochondrial fission protein Drp1, alleviates mitochondrial dysfunction and cell injury. To our knowledge, our research is the first to reveal that reduced expression of Mfn2 contributes to intestinal damage after DON exposure.

Considerable attention has been given to DON contamination in commonly consumed cereals and cereal’ by-products due to its threat to human and animal’ health [6, 25]. The intestine is the first defense to DON invasion, representing that the intestine can be exposed to higher contents of mycotoxins than other organs [26]. Swine is most sensitive to DON contamination, whose physiology is most similar to humans. After DON intake, it is firstly absorbed by small intestine and then entered into the systemic blood circulation. Increasing evidence demonstrated that DON could destroy intestinal structure and cause barrier impairment [27]. However, the underlying mechanism still remains poorly unknown. Clarifying the mechanism of intestinal damage by DON will provide important insights to prevent the adverse effects of DON on gut health of human or animals. At present, the intestinal toxicity of DON has been well-documented, with reported effects including epithelial damage, reduced barrier function, gut microbiota disruption, and inflammatory response induction, consequently leading to diverse cell death mechanisms [6, 7]. We further verified whether DON exposure had negative effects on intestinal structure in piglets. In the current study, dietary DON and DON gavage caused jejunal damage in piglets, such as epithelium lifting and villus atrophy. Further analysis showed that dietary DON and DON gavage resulted in increased inflammatory cell infiltration and inflammatory cytokines release. The increase of neutrophils often indicates infection and injury occurrence in gut. This is in accordance with previous study, which reported that DON impaired immune functions of neutrophils in pigs [28]. Our data also demonstrated that dietary DON increased IL-6 content in serum and DON gavage increased TNF-α and IL-6 contents in serum. The discrepancies in inflammation might be related to the different concentration and time period of DON exposure to piglets. In acute DON exposure model, piglets were given high dose of DON when they are fasted for 12 h. The DON rapidly enter the small intestine, and the peak absorption occurs earlier than repeated exposure in low dose by feeding contained diet. Therefore, oral administration of DON led to a more rapid intestinal inflammatory response. The continuous and repeated low-dose stimulation of DON in the diet generated a certain degree of immune tolerance in the body, so the inflammatory response is not as intense and extensive as that in the acute DON exposure model [29]. Our results are supported by previous reports in pigs [30, 31]. These data demonstrated that DON exposure led to intestinal damage in weaning pigs. To better verify the role of DON on cell damage, we treated IPEC-1 cells, which were originally derived from piglets, with DON challenge. We revealed that DON led to cell damage in IPEC-1 cells, which is consistent with the results of our animal trials. This is also supported by our previous report that DON caused IPEC-1 cell damage [23, 32]. Generally, these results indicated that DON led to compromised intestinal structure in piglets and IPEC-1 cells.

Mitochondria, as important dynamic organelles, constantly undergo fission and fusion, which is important for maintaining general physiological functions of cells [9, 33]. The mitochondrial dynamics is important for sustaining mitochondrial structure and function because it modulates mitochondrial content exchange between individual mitochondria and interaction between mitochondria and cytosol [33, 34]. Accumulating evidence has indicated that disturbance of mitochondrial fusion/fission triggers mitochondrial dysfunction, which subsequently causes cell death and diseases [9, 35]. Mitochondrial dynamic equilibrium is delicately regulated by mitochondrial fusion proteins (Mfn1, Mfn2 and Opa1) and fission proteins (Drp1 and Fis1) [9]. We guessed that DON-caused intestinal damage in piglets maybe associate with mitochondrial disorders. Therefore, we further explored the effect of DON on mitochondrial fission/fusion proteins. We uncovered that dietary DON and DON gavage both downregulated protein expression of Drp1 and Mfn2 in jejunum of piglets. In addition, DON exposure led to reduced protein expression of Drp1 and Mfn2 in IPEC-1 cells. Until now, the research about the harmful effect of DON on mitochondrial fission/fusion dynamic in intestine are very rare. Similar to our data, Ren et al. [36] reported that DON downregulated protein expression of Mfn1, Mfn2 and Opa1 in pig spleen lymphocytes, which suggested DON inhibited mitochondrial fusion. Haung et al. [37] also demonstrated that indoxyl sulfate inhibited protein expression of Drp1 in mice. During mitochondrial fission/fusion process, mitochondrial networks are dramatically reorganized, which are coordinated with cell apoptosis. In our study, we observed that DON exposure induced cell apoptosis indicated by increasing protein expression of caspase 3 both in piglets and IPEC-1 cells. This is supported by Kang et al. [38] who found that DON induced apoptosis in IPEC-J2 cells. Together, our results demonstrated that DON induced disorders of mitochondrial fission/fusion, and resulted in cell apoptosis. These results supported the conclusion that abnormal mitochondrial dynamics maybe involve in DON-induced intestinal injury in piglets.

To further explore whether DON-induced intestinal damage is partially due to abnormal expression of mitochondrial fission/fusion proteins, Drp1 and Mfn2 overexpression plasmids were used. Interestingly, in our present study, we found that Mfn2, but not Drp1 overexpression rescued cell apoptosis, mitochondrial dysfunction and cell damage after DON exposure. In agreement with our findings, Dong et al. [39] reported that enhancing mitochondrial fusion by Mfn2 overexpression plasmid restored PBDE-47-caused mitochondrial dynamic, morphological and functional damage in PCl2 cells. To further verify whether reduced Mfn2 contributed to intestinal damage in IPEC-1 cells, a mitochondrial fusion promoter M1 [39] was used to restore the mitochondrial fusion protein of Mfn2 in our subsequent experiment. As expected, consistently with Mfn2 overexpression, M1 effectively suppressed DON-induced decrease of Mfn2, which suggested that M1 rescued mitochondrial fusion protein expression after DON exposure. Our results were supported by Surinkaew et al. [40] who found that M1 alleviated cardiac ischemia/reperfusion-induced brain injury by increasing protein expression of Mfn2. We subsequently tested if promoting mitochondrial fusion by M1 could alleviate cell apoptosis. Expectedly, M1 inhibited cell apoptosis in IPEC-1 cells, indicating that Mfn2 had a positive role in restraining cell apoptosis after DON exposure. Similar with our data, Ding et al. [41] found that restoration of Mfn2-mediated mitochondrial fusion reduced cellular apoptosis in doxorubicin-treated cardiomyocytes. M1 also was reported to mitigate cell apoptosis in TPHP induced-damage in testicular Leydig cells and TM3 cells [42, 43]. Subsequently, we explored the role of Mfn2 on DON-caused mitochondrial dysfunction and cell injury. We found that M1 could reverse the decrease of MMP, which implied that increasing expression of Mfn2 could preserve mitochondrial function after DON exposure. Additionally, we uncovered that M1 prevented DON-caused cell damage and barrier dysfunction. Similarly, it was also reported that M1 or Mfn2 overexpression restored PBDE-47-caused morphological and functional damage, promoted neuronal survival in PC12 cells [39]. Until now, the research about the role of mitochondrial fission or fusion proteins in intestinal injury is very lacking. Based on our results, we reveal for the first time that abnormal protein expression of Mfn2 participates in DON-induced compromised intestinal structure and function in weaned pigs.

## Conclusions

In summary, our study demonstrated a critical role of Mfn2, but not Drp1 in DON-induced intestinal damage. Promoting protein expression of Mfn2 mitigated DON-induced damage of intestinal structure and function. This work provides novel insights into pathogenesis of mycotoxin toxicity, highlighting that Mfn2 may be a novel therapeutic candidate for intestinal damage after DON exposure in human or animals.

## Supplementary Information

Additional file 1. Table S1. The primer pairs of *Drp1* and *Mfn2* used by PCR. Fig S1. The overexpression (OE) efficiency of Drp1 and Mfn2 after transfection by plasmids in IPEC-1 cells. (A) *Drp1* mRNA expression after Drp1 OE plasmid transfection. (B) *Mfn2* mRNA expression after Mfn2 OE plasmid transfection.

Additional file 2: The original gel and blot images.

## Data Availability

The data for this study are available from the corresponding author upon reasonable request.

## Abbreviations

BW: Body weight
DON: Deoxynivalenol
Drp1: Dynamin related protein 1
FD4: Fluorescein isothiocyanate-labeled dextran 4 kDa
Fis1: Fission protein 1
HE: Hematoxylin and eosin
IBD: Inflammatory bowel disease
IPEC: Intestinal porcine epithelial cell line
LDH: Lactate dehydrogenase
Mfn1: Mitofusin 1
Mfn2: Mitofusin 2
MMP: Mitochondrial membrane potential
OE: Overexpression
Opa1: Optic atrophy protein 1
TEER: Transepithelial electrical resistance
