# Supplementary material for: Mitofusin 2 is required for preventing deoxynivalenol-induced porcine intestinal epithelial cell damage
Source: J Anim Sci Biotechnol. 2025 Dec 23;16:178. doi: 10.1186/s40104-025-01306-6 (PMC12723839; doi:10.1186/s40104-025-01306-6)
Supplement: Supplementary file 1 — Additional file 1. Table S1. The primer pairs of Drp1 and Mfn2 used by PCR. Fig S1. The overexpression (OE) efficiency of Drp1 and Mfn2 after transfection by plasmids in IPEC-1 cells. (A) Drp1 mRNA expression after Drp1 OE plasmid transfection. (B) Mfn2 mRNA expression after Mfn2 OE plasmid transfection. [file 40104_2025_1306_MOESM1_ESM.docx]

**Table S1** The primer pairs used by PCR

| Target | Primer sequence | Size, bp | GenBank number |
| --- | --- | --- | --- |
| *Drp1* | F: GTCGTGACTTGTCTTCTTCGTA  R: ACCTTGCCATCAGCCTCAG | 278 | XM_021092060.1 |
| *Mfn2* | F: CGCCAGTTTGTAGAGTACGC  R: CACAGGTGAGCAAAGGTTCC | 113 | XM_021095349.1 |
| *GAPDH* | F: CGTCCCTGAGACACGATGGT  R: GCCTTGACTGTGCCGTGGAAT | 194 | AF017079.1 |


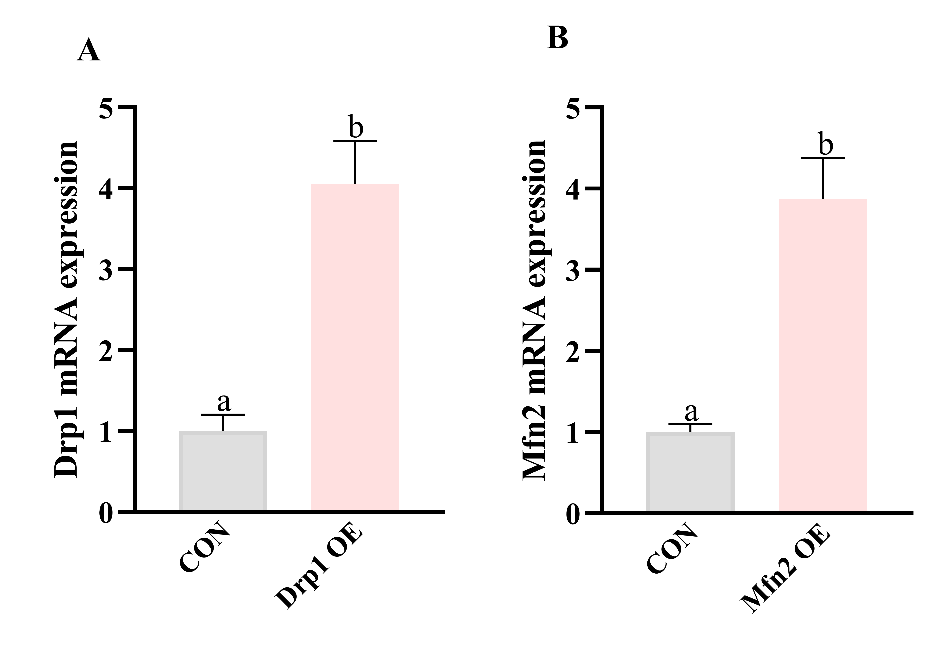


**Fig. S1** The overexpression (OE) efficiency of *Drp1* and *Mfn2* after transfection by plasmids in IPEC-1 cells. **A** *Drp1* mRNA expression after Drp1 OE plasmid transfection. **B** *Mfn2* mRNA expression after Mfn2 OE plasmid transfection. ^a,b^Means without a common letter differ (*P* < 0.05)
