# Supplementary material for: Mitofusin 2 is required for preventing deoxynivalenol-induced porcine intestinal epithelial cell damage
Source: J Anim Sci Biotechnol. 2025 Dec 23;16:178. doi: 10.1186/s40104-025-01306-6 (PMC12723839; doi:10.1186/s40104-025-01306-6)
Supplement: Supplementary file 2 — Additional file 2: The original gel and blot images. [file 40104_2025_1306_MOESM2_ESM.pdf]

Repeated DON exposure in pigs

Fig. 2A

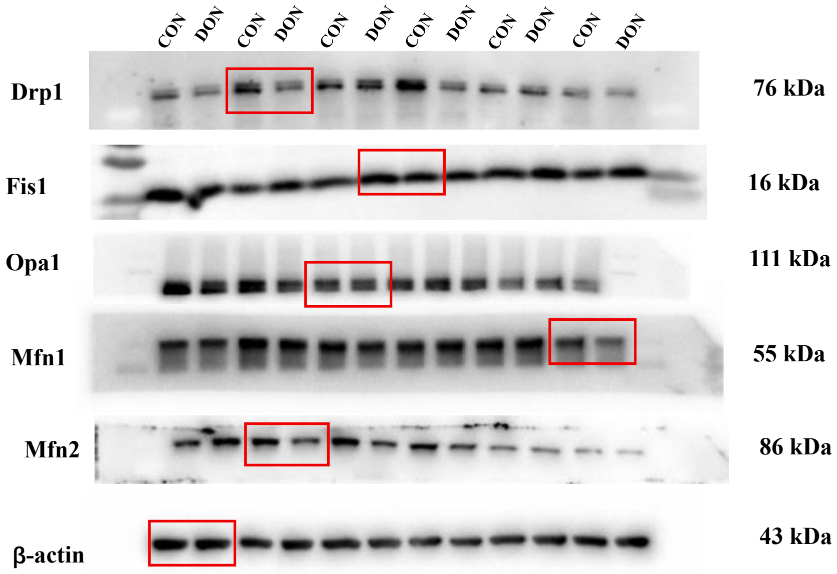

Fig. 2B

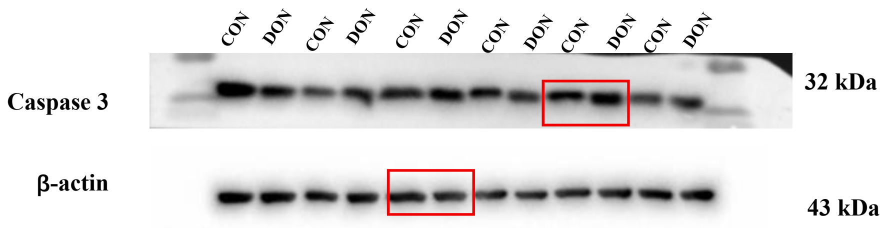

Acute DON exposure

Fig. 2C

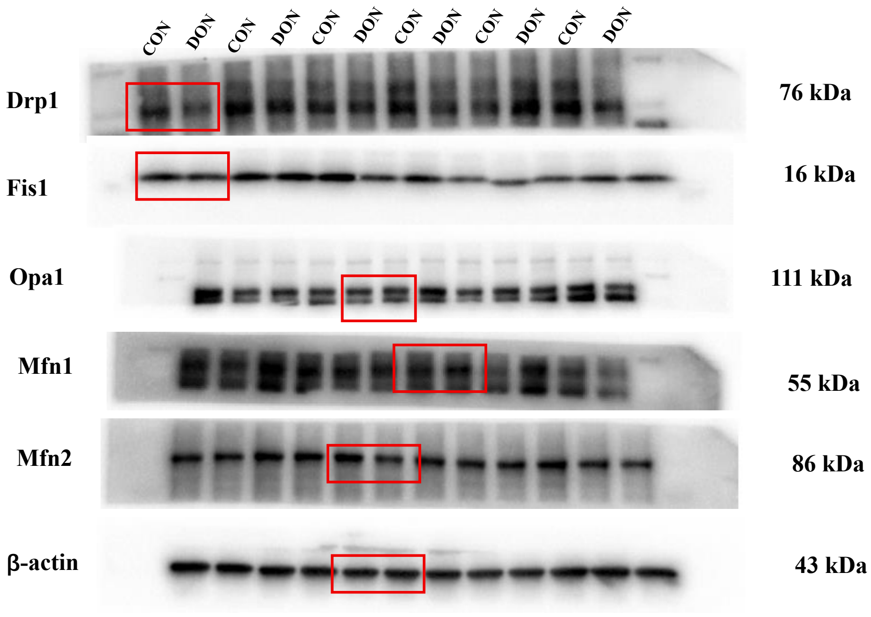

Fig. 2D

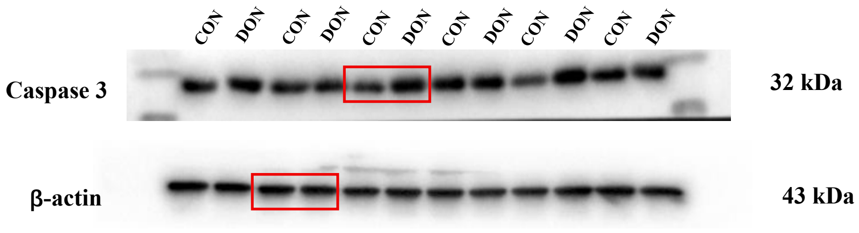

Fig. 3K

***In vitro* trials**

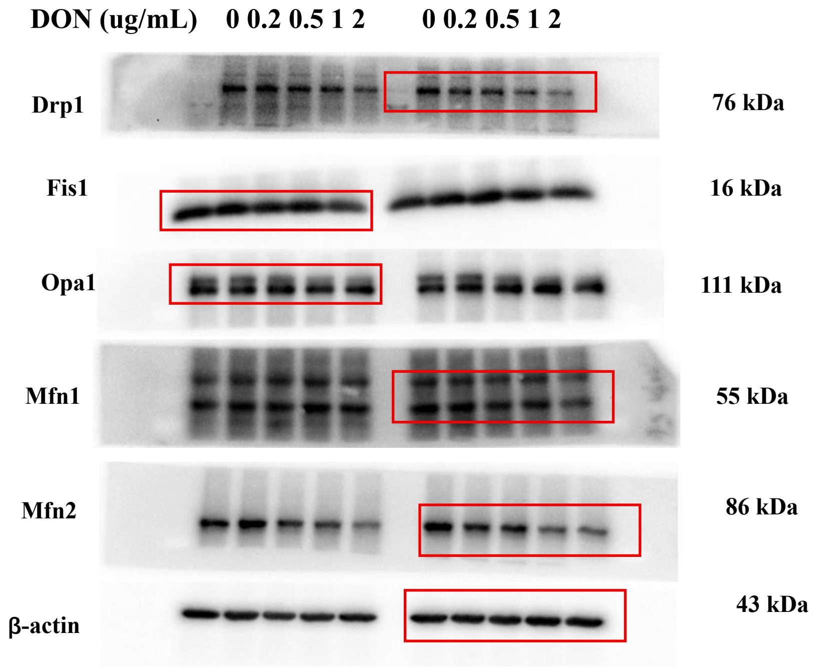

Fig. 3M

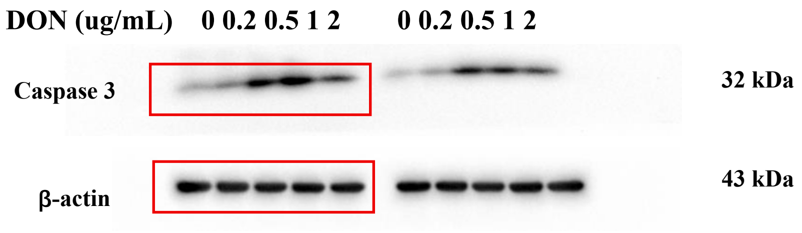

Fig. 4F

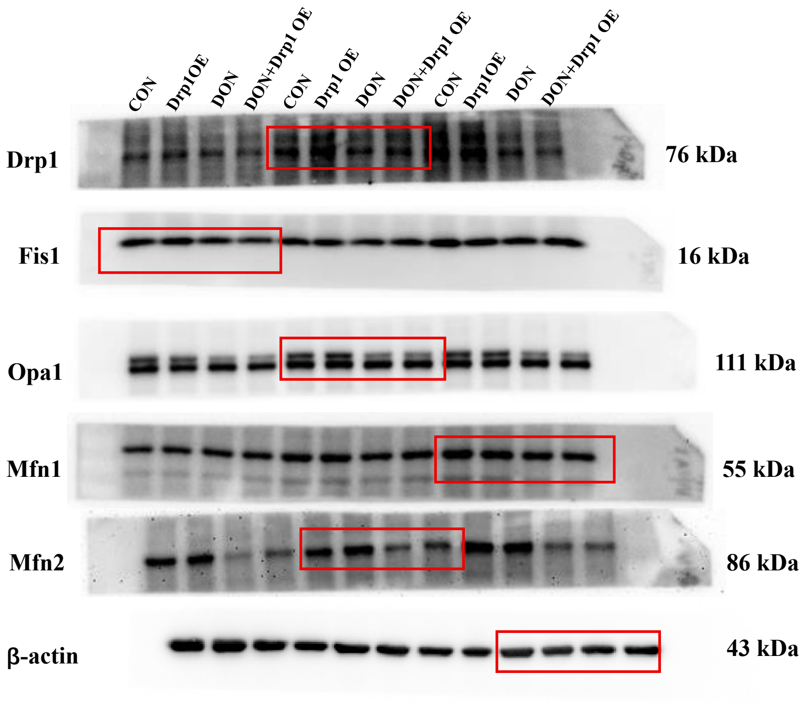

CON
Drp1OE
DON
DON+Drp1 OE
CON
Drp1 OE
DON
DON+Drp1 OE
CON
Drp1OE
DON
DON+Drp1 OE
Drp1
76 kDa
Fis1
16 kDa
Opa1
111 kDa
Mfn1
55 kDa
Mfn2
86 kDa
β-actin
43 kDa

Fig. 4H

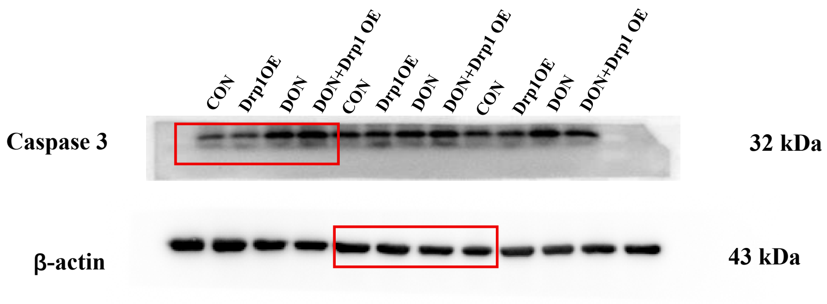

CON
Drp1OE
DON
DON+Drp1 OE
CON
Drp1OE
DON
DON+Drp1 OE
CON
Drp1OE
DON
DON+Drp1 OE
Caspase 3
32 kDa
β-actin
43 kDa

Fig. 5F

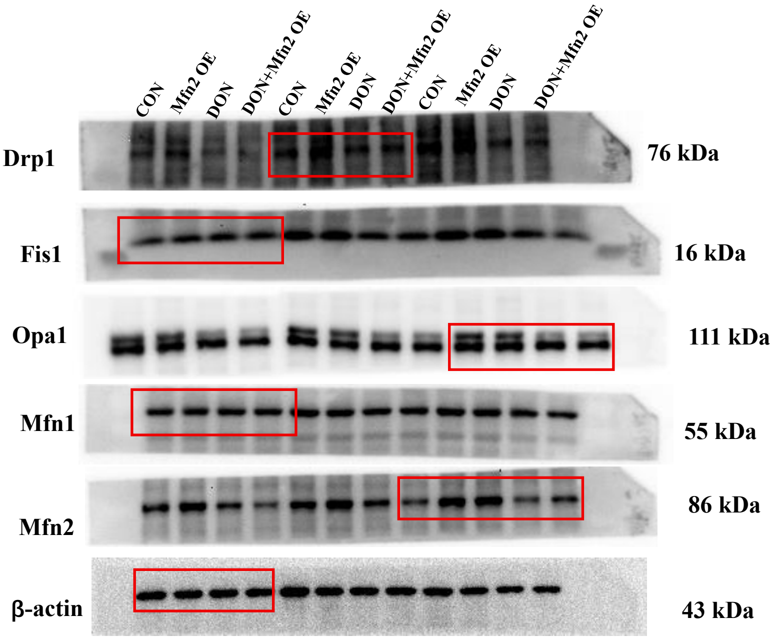
CON
Mfn2 OE
DON
DON+Mfn2 OE
CON
Mfn2 OE
DON
DON+Mfn2 OE
CON
Mfn2 OE
DON
DON+Mfn2 OE
Drp1
76 kDa
Fis1
16 kDa
Opa1
111 kDa
Mfn1
55 kDa
Mfn2
86 kDa
β-actin
43 kDa

Fig. 5H

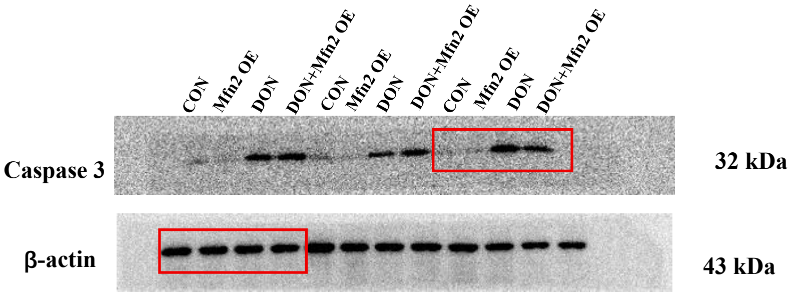
CON
Mfn2 OE
DON
DON+Mfn2 OE
CON
Mfn2 OE
DON
DON+Mfn2 OE
CON
Mfn2 OE
DON
DON+Mfn2 OE
Caspase 3
32 kDa
β-actin
43 kDa

Fig. 6F

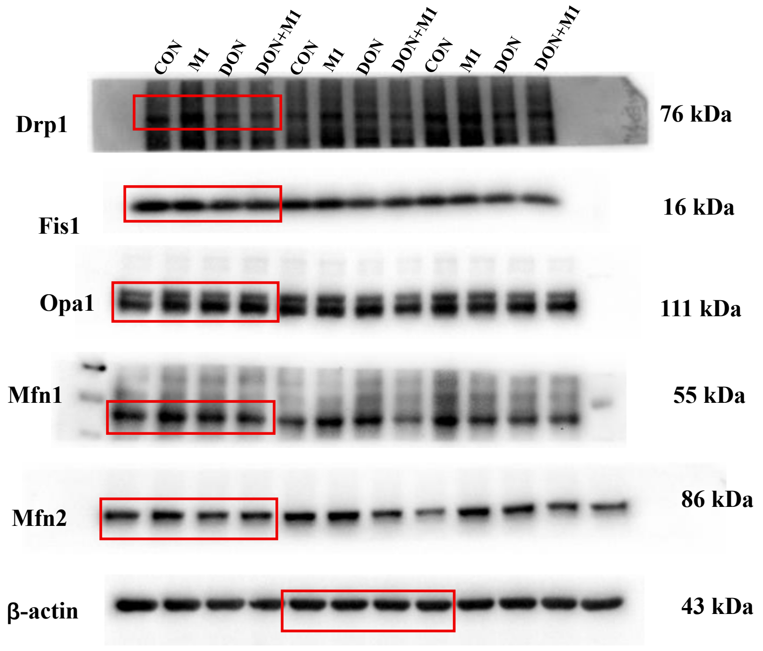
CON
M1
DON
DON+M1
CON
M1
DON
DON+M1
CON
M1
DON
DON+M1
Drp1
76 kDa
Fis1
16 kDa
Opa1
111 kDa
Mfn1
55 kDa
Mfn2
86 kDa
β-actin
43 kDa

Fig. 6H

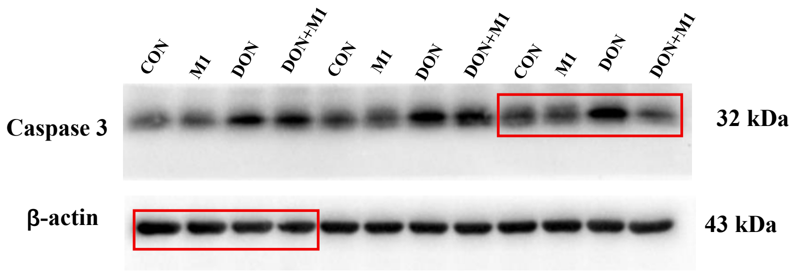
CON
M1
DON
DON+M1
CON
M1
DON
DON+M1
CON
M1
DON
DON+M1
Caspase 3
32 kDa
β-actin
43 kDa
